# Electromyographic investigation of unstable patella before and after its realignment operation

**DOI:** 10.4103/0019-5413.73662

**Published:** 2011

**Authors:** DD Baksi, AK Pal, DP Baksi

**Affiliations:** Department of Orthopedics, K.P.C. Medical College, Jadevpur, Kolkata, India; 1 Department of Orthopedics, Burdwan Medical College, Kolkata, India; 2 Department of Orthopedics, Medical College and Hospital, Kolkata, India

**Keywords:** EMG, patellar instabilities, realignment operation

## Abstract

**Background:**

Patellar dislocations are either due to superolateral contracture of the soft tissue or imbalance of the power between the vastus medialis (VM) and the vastus lateralis (VL). The imbalance of muscle power as an etiology of patellar dislocation has not been studied. Hence, we studied the recurrent, habitual and permanent dislocations of the patella with an electromyogram (EMG) of the vastus medialis, vastus lateralis, and pes anserinus, before and after realignment operations, to document the muscle imbalance and effectiveness of the realignment operation.

**Materials and Methods:**

An electromyographic investigation was carried out on the vastus medialis and vastus lateralis in nine recurrent, 20 habitual, and 13 permanent dislocations of the patella, before and after their realignment operations. Pes anserinus transposition, which acted as a medial stabilizer of the patella, was also investigated with an EMG study, to understand its role on patellar stability at 0°, 30°, 60°, 90°, 120°, 150°, and full flexion of the knee. The age of the patients varied from nine to 30 (mean 15) years. There were 24 males and 18 females. Twenty-six patellar dislocations were on the right and 16 were on the left side.

**Results:**

Electromyographic pictures reveal subnormal activity of the vastus medialis in all types of dislocations and similar activities of the vastus lateralis in permanent and habitual dislocations recorded pre operatively, which recovered to almost normal values postoperatively, at the mean one-year follow-up. Pes anserinus, which was used for medial stabilization of the patella after its realignment, maintained normal EMG activity before and after the operation.

**Conclusion:**

This study is significant for understanding the imbalance of muscle activities in patients with an unstable patella, which can be rectified without recurrence after pes anserinus transposition.

## INTRODUCTION

The instability of the patella may be classified as recurrent or habitual, and permanent subluxation or dislocation that often occur in the lateral direction of the knee. Dislocation is recurrent where it is episodic and habitual when it occurs commonly during each flexion movement of the knee. Permanent variety means dislocation persists in all positions of the knee. This may be congenital, due to myodysplasia,^1^ or acquired as a result of progressive superolateral muscle contracture, or it may be idiopathic. In a habitual dislocation, there is a lesser degree of superolateral contracture, similar to the acquired variety of permanent dislocation. In both these types, the contracture is the primary pathology, whether it is idiopathic or acquired, due to injection fibrosis;^2^ whereas, medial laxity or weakness in the medial stabilizers of the patella is secondary. In recurrent dislocation, the medial stabilization of the patella is poor because of weakness of the vastus medialis or its dysplasia, generalized joint laxity or posttraumatic medial capsular laxity, and there is no contracture primarily lateral to the patella.^3^–^6^

The superolateral contractures have been identified and extensively studied in a cadaveric study,^7^ whereas, in patellar dislocations, the muscle imbalance has not yet been adequately studied and investigated with electromyography (EMG) except in subluxations of the patella.^8^ Moreover, there is no substantial proof of muscle imbalance as an etiology of patellar dislocations in the literature, using EMG.

It is important to document imbalance in muscle power between VM and VL preoperatively as also the pattern of imbalance, to define the deforming force. This will make our understanding clear whether VL alone or VL along with other components of the quardriceps need to be cut.

**Figure 1:**
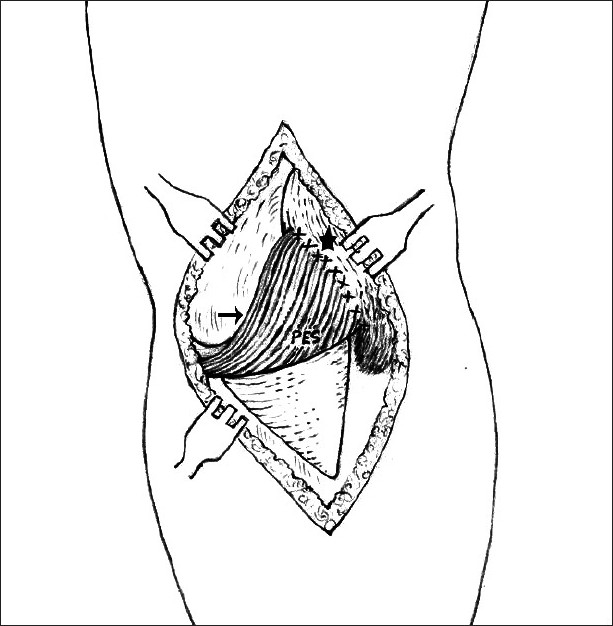
Line diagram shows pes anserinus (solid arrow) transposition, medial stabilizer of patella (star)

The superolateral contracture release of the patella, to enable the patella to be reduced in the intercondylar groove, in a fully flexed position of the knee, followed by the medial stabilization of the patella by pes anserinus transposition [Figure 1], was advocated for habitual and permanent dislocation,^9^,^10^ while for the treatment of the recurrent dislocation of the patella, its medial stabilization by pes anserinus transposition to the patella was performed, as there was no such lateral contracture.^9^,^10^

Hence, we studied the recurrent, habitual, and permanent dislocation of the patella with EMG of the vastus medialis, vastus lateralis, and pes anserinus, before and after realignment operations, to document muscle imbalance and effectiveness of the realignment operation. We compared the EMG activities of VL, VM, and Pes anserinus, to find out the possibility, pattern of muscle imbalance, and improvement in imbalance after the realignment operation compared to the preoperative level, to document the effectiveness of the pes anserinus as a medial stabilizer of the patella, to prevent its recurrence.

## MATERIALS AND METHODS

We conducted an EMG study of a total of 42 dislocations of the patella, which included nine recurrent, 20 habitual, and 13 permanent dislocations during the last six years.

All cases included here had unilateral affections; hence, the opposite normal knee was used as the control. The mean age of patients was 15 years (range 9–15 years). The mean age of the patients with recurrent dislocation was 17 years (range 14–38 years), with permanent dislocations was 14 years (range 4–30 years) and with habitual dislocations was 15 years (range 5–26 years). Patients less than nine years of age were excluded as they were unable to respond to commands during the EMG procedure. The patients who had congenital dislocation, bilateral affection, and age below nine years and above 30 years were excluded. There were 24 males and 18 females; 26 patellar dislocations were on the right and 16 were on the left side. Preoperatively, all the patients had full range of motion and were subjected to adequate superolateral contracture release in habitual (n=20) and permanent (n=13) dislocations, till the patella could be reduced in the intercondylar groove followed by pes anserinus transposition. Only pes anserinus transposition was needed in recurrent dislocation where there was no contracture (n=9) lateral to the patella. Postoperatively, all the patients had quadriceps lag, from which they recovered in two to five months (mean 3.2 months). The EMG study was done only after complete recovery of the full range of motion. The patients had minor complications like saphenous nerve irritation in two and temporary anterior knee pain from retropatellar degenerative change in two patients, which did not influence the final functional outcome. The surface electrodes were placed over the muscle bellies of the corresponding muscles, while the patient sat, lay down, and performed voluntary extensions of the knee against gravity with no resistance at 0°, 30°, 60°, 90°, 120°, 150°, and in full flexion of the knee. The different grades of EMG activity were measured by observing the character of the EMG graph patterns in the different limbs, compared to the normal EMG graph pattern of the muscles on the normal side. Therefore, if the EMG graph pattern was similar to that of the normal side it was considered normal activity and if no pattern was observed with an isoelectric line the activity was considered nil or subnormal.

The EMG evaluation of the vastus medialis, vastus lateralis, and pes anserinus muscles was performed using bipolar surface electrodes in a Dantec Neuromatric 2000 C Electromyograph with a calibration of 10 ms / division and 0.5 mv / division, before and after recovery of the quadriceps lag following the realignment operations. Preoperative EMG was performed on all the patients. EMG studies were repeated two-to-three times, post-operatively for one-year, when no further significant change in EMG activities were observed. The first EMG was performed just after recovery of the quadriceps lag, second EMG after three months of the first EMG, and finally at one year. The EMG studies of the corresponding muscles of an individual were done at similar sites on both the normal as well as the pathological sides of the knee, to compare their activities in different flexion positions of the knee, as stated earlier.

## RESULTS

**Figure 2:**
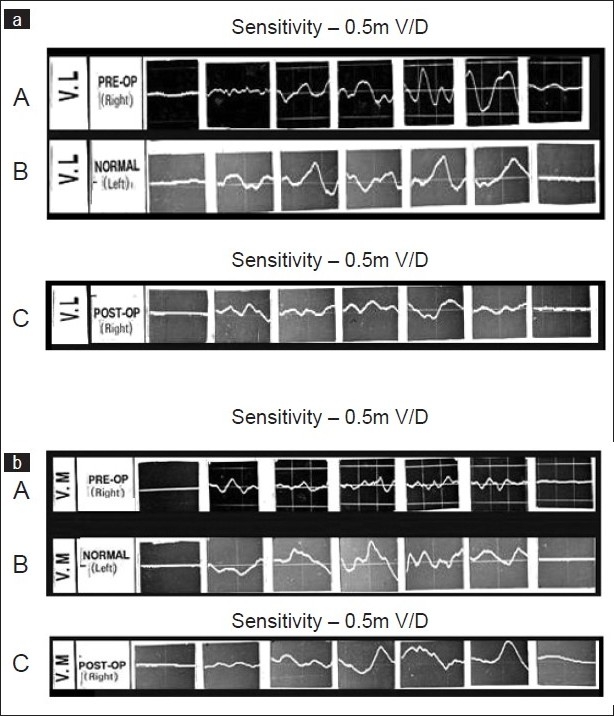
E.M.G pattern of Vastus lateralis (VL) (Figure 2a A.B.C) and vastus medialis (VM) (Figure 2b A.B.C) in cases with recurrent dislocations of right patella. There are seven blocks of EMG each recorded at 0°, 30°, 60°, 90°, 120°, 150°, 160° (full flexion) of knee flexion. The graph A of 2a and 2b shows the normal EMG activity of VL and VM respectively of normal left limb. Pre operative EMG of VL (B of 2a) and VM (B of 2b) shows subnormal activity of VM and near normal activity of VL suggesting imbalance of muscular activity which become normal after operation as suggested by normal EMG activity of VL (C of 2a) and VM (C of 2b) at one year follow up after pes-anserinus transposition

**Figure 3:**
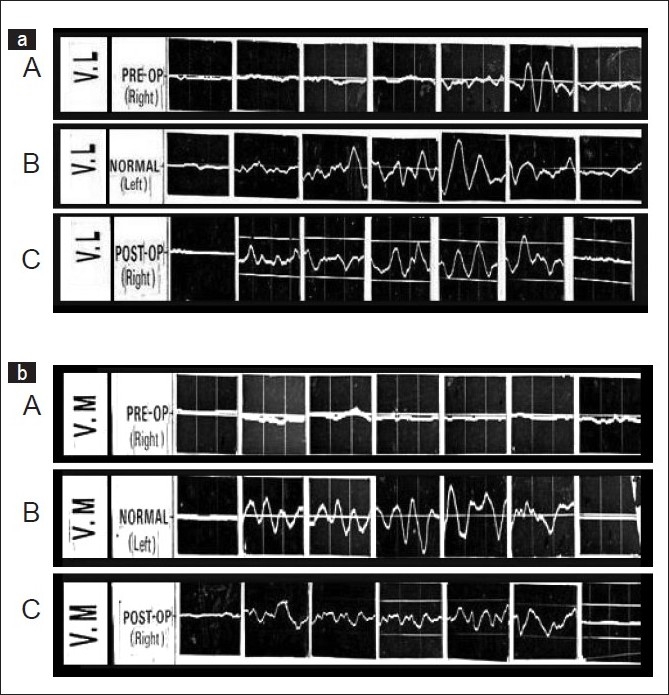
EMG pattern of vastus lateralis (VL) (Figure 3a A.B.C) and vastus medialis (VM) (Figure 3b A.B.C) in permanent dislocation of right patella. There are seven blocks of EMG each recorded at 0°, 30°, 60°,90°,120°,150°,160° (full flexion) of knee flexion. The graph A of 3a and 3b shows the normal EMG activity of VL and VM respectively of normal left limb. Pre operative EMG of VL (B of 3a) and VM (B of 3b) shows subnormal activity of both except near normal activity of VL at 120° to 150° range of terminal flexion suggesting imbalance of muscular activity which become normal after operation as suggested by normal EMG activity of VL (C of 3a) and VM(C of 3b) at one year follow up after pes-anserinus transposition

In case of recurrent dislocation, preoperative EMG study showed that VL activity remained good, and the VM activity was subnormal throughout the arc [Figure 2]. In cases with permanent dislocation [Figure 3] the preoperative activity of VM and VL were almost nil or subnormal except at 120° to 150° range of flexion, when the VL activity was demonstrated significantly, like a flexor of the knee [Figure 3].

**Figure 4:**
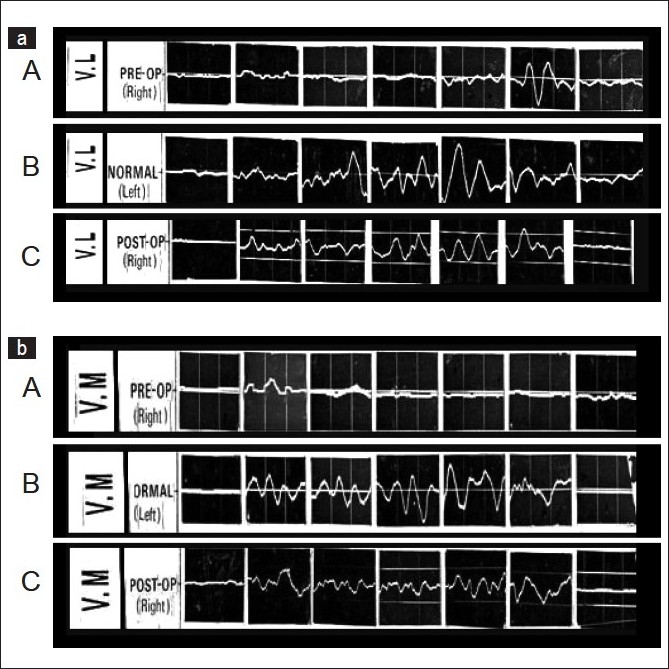
E.M.G pattern of Vastus lateralis (VL) (Figure 4b A.B.C) and vastus medialis (VM) (Figure 4b A.B.C) in cases with habitual dislocations of right patella. There are seven blocks of EMG each recorded at 0°, 30°, 60°, 90°, 120°, 150°, 160° (full flexion) of knee flexion. The graph A of 4a and 4b shows the normal EMG activity of VL and VM respectively of normal left limb. Preoperative EMG activity of VL (B of 4a) and VM (B of 4b) shows subnormal activity of the VM throughout the arc and near normal activity of VL at 30o which is the angle of dislocation in this case and again between 120° to 150° suggesting imbalance of muscular activity which become normal after operation as suggested by normal EMG activity of VL (C of 4a) and VM (C of 4b) at one year follow up after pes-anserinus transposition

In cases with habitual dislocation of the patella [Figure 4] there was subnormal activity of the VM throughout the arc and normal activity of VL up to the angle of dislocation, usually between 30° and 40° flexion of the knee joint, and then the activity deteriorated, except during 120°–150° knee flexion, like a flexor of the knee.

Therefore, a preoperative EMG study clearly suggests that imbalance of muscle power exists and subnormal VM activity remains a significant factor in all the dislocations, where it stands out as the sole defect in the recurrent type, where no contractures superolateral to the patella exist. On the other hand, VM being a weaker counterpart, should neither be selected as a medial stabilizer of the patella during the realignment operation, nor should be included in the superolateral contracture release.

**Figure 5:**
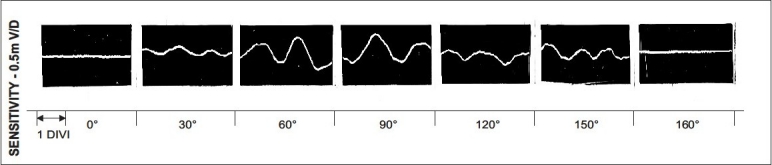
EMG pattern of pes muscles after its transposition. There are seven blocks of EMG each recorded at 0°, 30°, 60°, 90°, 120°, 150°, 160° (full flexion) of knee flexion. This post operative EMG study of the pes anserinus showed that its activity continued throughout the range of flexion of the knee, reaching its highest level between 60° and 120° flexion, and then it diminished as flexion increased. Similar EMG graph of Pes Anserinus was recorded in every follow up after its transposition even at one year follow up as above

Vastus lateralis showed normal EMG activity at a mean three-month follow-up after the realignment operation, where the quadriceps lag disappeared in all types of patellar dislocations; whereas, the VM activity started to improve in three to five months, and took several months to slowly and completely recover in the recurrent, and nearer to normal in the habitual as well as permanent dislocations — around one year. Following this the EMG activity did not show further improvement. From the postoperative EMG study, it was evident that the VM weakness was not permanent, but it was during the early postoperative period when the VM activity was subnormal (window period). There was a requirement for an active medial stabilizer of the patella to keep it in the intercondylar groove and prevent a recurrence of the dislocation. A postoperative EMG study of the pes anserinus showed that its activity continued throughout the range of flexion of the knee, reaching its highest level between 60° and 120° flexion, and then it diminished as flexion increased [Figure 5]. Palpable contraction of the transposed pes anserinus increased up to 90° of knee flexion, which was correlated and confirmed electromyographically. Similar EMG activity of the pes anserinus was seen to be maintained throughout the follow-up period. Hence, the efficacy of pes anserinus has proved to be an active restraint to lateral patellar dislocations among the dynamic structures, on the medial side.

## DISCUSSION

In a preoperative study, we have to see whether the imbalance of the muscle power exists in such a condition; if so, what is the pattern of imbalance and which muscle is maximally contributing as a deforming force. Also during the release of the superolateral contractures, whether only the vastus lateralis (VL) alone or VL along with other components of the quadriceps, including the vastus medialis (VM) should be cut^11^ or not.

For habitual and permanent dislocations, the superolateral contracture release of the patella was performed judiciously, to enable the patella to be relocated in the intercondylar groove in a fully flexed position of the knee, followed by medial stabilization of the patella by pes anserinus transposition [Figure 1],^9^,^10^ to prevent its further dislocation. In the treatment of recurrent dislocation of patella, as there is no such lateral contracture, its medial stabilization is performed by pes anserinus transposition to the patella.^9^,^10^

This EMG study proves that muscle imbalance is the cause of patellar instability, where it shows uniform inference of weakness of the VM in all types of dislocations. Vastus medialis prevents lateral dislocation of the patella by resisting the subluxatory pressure created by valgus angulations of the knee and vastus lateralis.^12^–^14^ Any change whether congenital dysplasia, post traumatic or idiopathic, or injection fibrosis in the muscle balance between the vastus medialis and vastus lateralis, is bound to affect the patellofemoral joint. According to the severity of disorders, recurrent, habitual, and permanent dislocations of the patella are the clinical presentations.

In recurrent dislocation of the patella, the preoperative diminished activity of the VM may be due to its dysplasia or inherent weakness, which has been noted where there is no deforming factor like a contracture lateral to the patella. In all types of dislocations of the patella even after their realignment in the intercondylar groove after superolateral contracture release, there is a tendency for the patella to move laterally because of the existing weakness in the VM for a considerable period of time (window period), before near normal recovery at around the one-year follow-up. It is suggested to continue the vigorous postoperative rehabilitation program for the initial postoperative period till adequate recovery of the muscle power of VM. Therefore, strengthening the VM is also essential along with the rehabilitation of pes anserinus, to prevent a recurrence. Moreover, in that window period, an EMG study of the pes anserinus proved its effectiveness as a dynamic medial stabilizer of the patella and prevented the recurrence of dislocations. Pes anserinus [Figure 1] acts as an unstretchable active sling, which retains its elasticity and physiological strength, because the blood and nerve supplies to the proximal muscle fibers are not disturbed.^9^,^10^ The wide tendon gives a broad base anchorage. The inferior tendons are prised up to a superior position after transposition, but are continuous proximally with the sartorius, gracilis, and semitendinosus muscles. In an extended position of the knee, the new tendinous insertion is at an angle [Figure 1], thus reducing the pull on the patella. In the flexed position, which favors patellar dislocation, however, when this angle is straightened there is a direct medial pull on the patella [Figure 1], which is correlated by the EMG studies [Figure 5]. In this way, the sling becomes more active in the flexed position of the knee, when recurrent and habitual dislocations usually occur.

Moreover, as the VM is weak preoperatively in all types of dislocations of the patella, it should neither be selected for reconstruction of the medial stabilization of the patella as done by Madigan *et al*.^15^ nor should it be included in the inverted V-Y plasty of the quadriceps in habitual dislocations of the patella in flexion, as done by Bergman *et al*.,^11^ which will make the VM more weak.

## CONCLUSION

The EMG study is valuable in recognising the pattern of muscle imbalance, before and after realignment operations and duration and pattern of recovery after alignment. The VM and VL showed subnormal activity preoperatively which recovered to normal in postoperative period. Pes anserinus showed normal EMG activity before and after realignment operation. It also explains the effectiveness of pes anserinus transfer in rectifying preexisting muscle imbalance associated with different patellar dislocations.
